# Synergistic association between underweight and type 2 diabetes on the development of laryngeal cancer: a national population-based retrospective cohort study

**DOI:** 10.1186/s12885-022-09403-9

**Published:** 2022-03-29

**Authors:** Oh. Hyeong Lee, Yong-Moon Park, Seung-Hyun Ko, Kyuna Lee, Yeonji Kim, Kyungdo Han, Jung-Hae Cho

**Affiliations:** 1 Department of Otolaryngology-Head and Neck Surgery, St. Vincent’s Hospital, College of Medicine, The Catholic University of Korea, Suwon-si, Republic of Korea; 2 Department of Epidemiology, Fay W. Boozman College of Public Health, University of Arkansas for Medical Sciences, Little Rock, AR USA; 3 Division of Endocrinology & Metabolism, Department of Internal Medicine, College of Medicine, The Catholic University of Korea, Seoul, Republic of Korea; 4 Department of Biostatistics, College of Medicine, The Catholic University of Korea, Seoul, Republic of Korea; 5 Department of Statistics and Actuarial Science, Soongsil University, Seoul, Republic of Korea

**Keywords:** Laryngeal cancer, Body mass index, Underweight, Type 2 diabetes, Diabetes mellitus, Cohort, Risk factors

## Abstract

**Background:**

Although cigarette smoking is the most significant risk factor for laryngeal cancer, other risk factors might also be associated with the development of laryngeal cancer. We investigated whether underweight and type 2 diabetes are associated with laryngeal cancer in a Korean population.

**Methods:**

A total of 9,957,059 participants (≥20 years) without prior history of cancer who underwent a National Health Insurance Service health checkup in 2009 were followed up until December 31, 2018. Newly diagnosed laryngeal cancer was identified using claim data, and underweight was defined as body mass index (BMI) < 18.5 kg/m^2^. A Cox proportional-hazards models with multivariable adjustment were used to estimate hazard ratios (HRs) and corresponding 95% confidence intervals (95% CIs).

**Results:**

During the median follow-up period of 8.3 years, 3504 cases of laryngeal cancer occurred. Underweight was associated with increased risk of laryngeal cancer after adjusting for potential confounders (HR: 1.43, 95% CI: 1.22–1.69) compared to those who were not underweight. Underweight and type 2 diabetes were synergistically associated with higher risk of laryngeal cancer (HR: 2.33, 95% CI: 1.54–3.51), compared to those without either condition. This relationship was stronger in those with an age < 65 years (HR: 3.33, 95% CI: 1.88–5.87) and alcohol consumption (HR: 2.72, 95% CI: 1.64–4.53).

**Conclusions:**

These results suggest that underweight may be a significant risk factor for laryngeal cancer and that underweight and type 2 diabetes might synergistically increase the risk of laryngeal cancer.

## Background

Over the past 20 years, the smoking rate among men decreased by half from 66.3% in 1998 to 36.7% in 2018 due to the influence of a national anti-smoking campaign in Korea [1]. On the other hand, the female smoking rate slightly increased from 6.5% in 1998 to 7.5% in 2018. Smoking is the single greatest risk factor for head and neck cancer (HNC), especially laryngeal cancer. Annually, over 184,000 people are newly diagnosed with laryngeal cancer, which is the 2nd most common cancer of the head and neck worldwide [2]. More than 1000 patients were newly diagnosed with laryngeal cancer in Korea in 2020 [3]. Although the smoking population is continuously decreasing, the global incidence of laryngeal cancer is not decreasing [4]. This trend suggests that there may be other potential risk factors for laryngeal cancer.

According to changes in lifestyle and dietary habits, and steady increases in the rates of chronic metabolic diseases such as obesity and diabetes mellitus (DM) during the last several decades, some metabolic parameters have been suggested as risk factors for the development of cancers [5, 6]. In general, DM and obesity are important contributing factors for solid organ cancers, including liver, kidney, colorectal, and pancreatic cancers [7–10].

It has been documented that body mass index (BMI) is inversely associated with the development of oral cancer, lung cancer, and esophageal squamous cell carcinoma, which are all closely related to tobacco smoking [11, 12]. Previous studies have found that people with a BMI < 18.5 kg/m^2^ experience a higher risk of HNC than people with a normal BMI (18.5–25.0 kg/m^2^) [12–15]. According to studies reporting an inverse relationship between BMI and HNC risk, similar results were confirmed in only a small number of patients with laryngeal cancer in populations from China and the Netherlands with short follow-up periods [13, 16].

DM is also known to be associated with the development of HNC, especially oral and nasopharyngeal cancer [17, 18]. In Korea, approximately half of patients with DM (53.2%) are obese [19], and cancer risk increases in patients with DM, especially in obese subjects [7]. However, this association has not yet been confirmed in individuals with HNC.

In this Korean nationwide population-based study, we aimed to investigate the association between BMI and laryngeal cancer risk and whether this association is mediated by type 2 diabetes.

## Methods

### Data source and study population

**Fig. 1:**
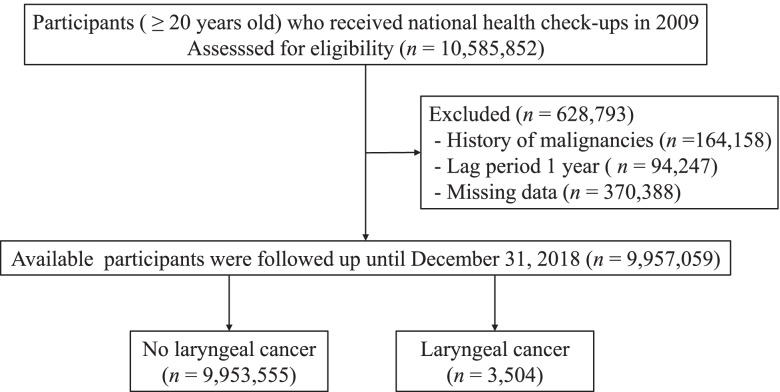
Flow chart of the cohort selection

In this retrospective cohort study, we used the Korean National Health Insurance System (NHIS) database, which is government managed and the only insurer providing regular health check-up programs to the public in South Korea. Based on the law, the NHIS may perform compulsory financial collections from the insured. The data collected for this operation is called ‘qualification and contribution data’. Those enrolled in the health insurance service are recommended to undergo health check-ups at least biennially. In the present study, we included 10,585,852 subjects aged ≥20 years who underwent a health examination in 2009. Participants who were diagnosed with laryngeal cancer during the preceding year or within 1 year after enrollment were excluded. Of these subjects, we excluded 164,158 having prior malignancy and 370,388 with missing data (Fig. 1). This study was conducted according to the Declaration of Helsinki and approved by the Institutional Review Board of the Catholic University of Korea (IRB No. VC21ZISI0191). Because anonymized and deidentified information was used in the analyses, the requirement for informed consent was waived by the Institutional Review Board of the Catholic University of Korea (IRB No. VC21ZISI0191). The reporting of this study followed recommendations in the Reporting Studies Conducted using Observational Routinely collected health Data (RECORD) guidelines [20].

### Definition of laryngeal cancer

The primary outcome was newly diagnosed laryngeal cancer during the follow-up period. The diagnosis of laryngeal cancer was detected by surveillance of the disease code. Laryngeal cancers were coded as International Classification of Diseases (ICD-10) codes such as C101, C320, C321, C322, C323, C328 and C329. Because the Korean Health Insurance Review and Assessment Service reimburses all medical care expenses for confirmed cancer, all cancer patients are registered in the database. Therefore, we could include each newly diagnosed cancer patient in the whole South Korean population. The study population was followed from baseline at the index year to the date of cancer diagnosis, December 31, 2018, or death, whichever came first.

### Data collection

Standardized self-reported questionnaires were used to collect baseline data for the following variables: age (years), sex, cigarette smoking (never, ex and current), alcohol consumption (never, mild and heavy), and physical activity. Smoking status was divided into three groups: nonsmoker (less than 5 packs or 100 cigarettes in lifetime), ex-smoker (smoked more than 5 packs or 100 cigarettes in life but not currently smoking), and current smoker (smoked more than 5 packs or 100 cigarettes in life and still smoking) [21]. Alcohol consumption was categorized into none, mild (< 30 g/day ethanol) and heavy (≥30 g/day ethanol).

Regular physical activities were evaluated according to the weekly frequency of moderate- or vigorous-intensity physical activity. Income status was divided into four groups based on health insurance contributions: Q1 (the lowest quartile), Q2, Q3 and Q4 (the highest quartile).

BMI was calculated as weight in kilograms divided by height in square meters. BMI was categorized into five groups; underweight (< 18.5 kg/m^2^), normal weight (18.5–22.9 kg/m^2^), overweight (23–24.9 kg/m^2^), obese (25–30 kg/m^2^), and severely obese (≥30 kg/m^2^) according to World Health Organization (WHO) guidelines for the Asian-Pacific region [22]. Blood samples for the measurement of serum glucose, total cholesterol, triglycerides, high-density lipoprotein cholesterol, and low-density lipoprotein cholesterol levels were drawn after fasting overnight. The presence of type 2 diabetes was defined according to the following criteria: (1) at least one claim per year under ICD-10 codes E11–14 and at least one claim per year for antidiabetic drug prescription or (2) fasting glucose level ≥ 126 mg/dL [23]. Patients with type 1 diabetes who had claims under ICD-10 code E10 were excluded from this study. Hypertension was defined based on ICD-10 codes I10–I13 and I15 plus a history of antihypertensive drug prescription. Dyslipidemia was defined as a total cholesterol level ≥ 6.21 mmol/L (≥240 mg/dL) or the presence of one or more claims per year for antihyperlipidemic medications with ICD-10 code E78. Patients with an estimated glomerular filtration rate (eGFR) < 60 mL/min/1.73 m^2^ at the time of baseline evaluation were classified as having chronic kidney disease (CKD) using the Modification of Diet in Renal Disease (MDRD) equation [24]. Anemia was defined in accordance with the WHO criteria as hemoglobin < 13 g/dL in men and < 12 g/dL in women [25].

### Statistical analysis

Data are presented as the mean ± standard deviation, median value (interquartile range) or n (%). The incidence of primary outcomes was calculated by dividing the number of incident cases by the total follow-up duration (person-years). The χ^2^ test was used to determine differences in the proportions of categorical variables, and Student’s t test was used to evaluate differences between the means of continuous variables. Hazard ratios (HRs) and 95% confidence intervals (CIs) for the development of laryngeal cancer were calculated using a Cox proportional hazards model for the association of BMI and type 2 diabetes after adjusting for confounders. The *P* values provided are two-sided, with the level of significance at 0.05.

A multivariable-adjusted proportional-hazards model was applied. Model 1 was adjusted for age, sex. Model 2 was adjusted further for income status, smoking, alcohol consumption, and physical activity. We further adjusted for type 2 diabetes, hypertension, dyslipidemia, and anemia in model 3. Subgroup analyses were performed to determine whether the association between laryngeal cancer and underweight with type 2 diabetes varies according to participant characteristics. Statistical analyses were performed using SAS software (v. 9.4; SAS Institute) and a *p* value < 0.05 was considered significant.

## Results

During the mean follow-up duration of 8.2 ± 0.9 years, there were 3504 individuals with newly diagnosed laryngeal cancer among 9,957,059 participants (Fig. 1). Table 1 shows the baseline characteristics of participants according to the development of laryngeal cancer. Compared with the individuals without laryngeal cancer, those with laryngeal cancer were more likely to be male, older, lean, abdominal obesity, current smokers, and alcohol consumers and to have type 2 diabetes, hypertension, anemia, dyslipidemia, and higher fasting glucose. Income level or socioeconomic status was not different between the two groups.

**Table 1:** Baseline characteristics of the study population according to the development of laryngeal cancer

Parameter	Laryngeal cancer (−)	Laryngeal cancer (+)	***p***-value
	(***n*** = 9,953,555)	(***n*** = 3504)	
Age at enrollment, years			
Mean	46.83 ± 14	61.63 ± 9.94	<.0001
Categories, *n* (%)			<.0001
20–39	3,196,973 (32.12)	60 (1.71)	
40–64	5,503,198 (55.29)	2011 (57.39)	
≥ 65	1,253,384 (12.59)	1433 (40.9)	
Male, *n* (%)	5,494,878 (55.2)	3322 (94.8)	<.0001
Smoking, *n* (%)			<.0001
Nonsmoker	5,889,510 (59.17)	812 (23.17)	
Ex-smoker	1,422,335 (14.29)	863 (24.63)	
Current smoker	2,641,710 (26.54)	1829 (52.2)	
Alcohol consumption, *n* (%)			<.0001
Nondrinker	5,078,363 (51.02)	1298 (37.04)	
Mild drinker	4,075,549 (40.95)	1518 (43.32)	
Heavy drinker	799,643 (8.03)	688 (19.63)	
Regular physical activity, *n* (%)	1,795,502 (18.0)	782 (22.3)	<.0001
Low income (Q1), *n* (%)	2,133,192 (21.43)	766 (21.86)	0.54
Type 2 diabetes, *n* (%)	855,037 (8.59)	659 (18.81)	<.0001
Anemia, *n* (%)	1,072,898 (10.78)	422 (12.04)	0.02
Hemoglobin, g/dL	13.97 ± 1.6	14.38 ± 1.42	<.0001
Hypertension, *n* (%)	2,537,201 (25.49)	1687 (48.14)	<.0001
Dyslipidemia, *n* (%)	1,791,225 (18)	824 (23.52)	<.0001
Chronic kidney disease, *n* (%)	677,243 (6.8)	341 (9.73)	<.0001
Height, cm	163.99 ± 9.27	166.01 ± 6.52	<.0001
Weight, kg	64.03 ± 11.67	64.59 ± 10.15	0.01
BMI, kg/m^2^			
Mean	23.71 ± 3.45	23.38 ± 3.04	<.0001
Categories, *n* (%)			<.0001
< 18.5	365,663 (3.67)	155 (4.42)	
18.5–22.9	3,872,796 (38.91)	1429 (40.78)	
23–24.9	2,454,246 (24.66)	912 (26.03)	
25–29.9	2,905,612 (29.19)	932 (26.6)	
≥ 30	355,238 (3.57)	76 (2.17)	
Waist circumference, cm	80.24 ± 9.46	83.9 ± 8.14	<.0001
Fasting glucose, mg/dL	97.17 ± 23.8	104.19 ± 28.25	<.0001
Systolic blood pressure, mm Hg	122.4 ± 15.02	128.5 ± 16	<.0001
Diastolic blood pressure, mm Hg	76.3 ± 10.05	78.8 ± 10.08	<.0001
Total cholesterol, mg/dL	195.32 ± 41.39	194.06 ± 49.4	0.07
HDL cholesterol, mg/dL	56.5 ± 32.96	55.96 ± 41.93	0.33
LDL cholesterol, mg/dL	121.35 ± 216.89	111.58 ± 63.37	0.01

Data are mean SD or n (%)

*Abbreviations*: *BMI* Body mass index, *HDL* High-density lipoprotein, *LDL* Low-density lipoprotein

Table 2 shows the association between BMI and laryngeal according to adjusted regression models. The incidence rate (IR) of laryngeal cancer was highest in underweight (0.052 events per 1000 person-years). In fully adjusted Cox regression models adjusted for age, sex, income level, smoking, alcohol consumption, regular physical activity, type 2 diabetes, hypertension, dyslipidemia and anemia, underweight (BMI < 18.5 kg/m^2^) was significantly associated with increased risk of laryngeal cancer, compared to those with a BMI ≥18.5 kg/m^2^ (HR: 1.43, 95% CI: 1.22–1.69), and those with a BMI 18.5–22.9 kg/m^2^ (HR: 1.25, 95% CI: 1.06–1.48), respectively.

**Table 2:** Hazard ratios for developing laryngeal cancer according to BMI

	IR (per 1000 person-years)	HR (95% CI)		
		Model 1	Model 2	Model 3
**Underweight**				
< 18.5	0.052	**1.59 (1.35–1.87)**	**1.36 (1.16–1.60)**	**1.43 (1.22–1.69)**
≥ 18.5	0.042	1 (Reference)	1 (Reference)	1 (Reference)
**BMI, kg/m**^**2**^				
< 18.5	0.052	**1.35 (1.14–1.59)**	**1.23 (1.04–1.45)**	**1.25 (1.06–1.48)**
18.5–22.9	0.045	1 (Reference)	1 (Reference)	1 (Reference)
23–24.9	0.045	0.81 (0.74–0.88)	0.87 (0.80–0.94)	0.84 (0.78–0.92)
25–29.9	0.039	0.71 (0.65–0.77)	0.78 (0.72–0.85)	0.74 (0.68–0.81)
≥ 30	0.026	0.77 (0.61–0.96)	0.83 (0.66–1.05)	0.76 (0.60–0.96)

Model 1 was adjusted for age, sex

Model 2 was adjusted for age, sex, income status, smoking, alcohol consumption, and physical activity

Model 3 was adjusted for age, sex, income status, smoking, alcohol consumption, physical activity, type 2 diabetes, hypertension, dyslipidemia, and anemia

*Abbreviations*: *BMI* Body mass index, *CI* Confidence interval, *HR* Hazard ratio, *IR* Incidence rate (1000 person-year)

**Fig. 2:**
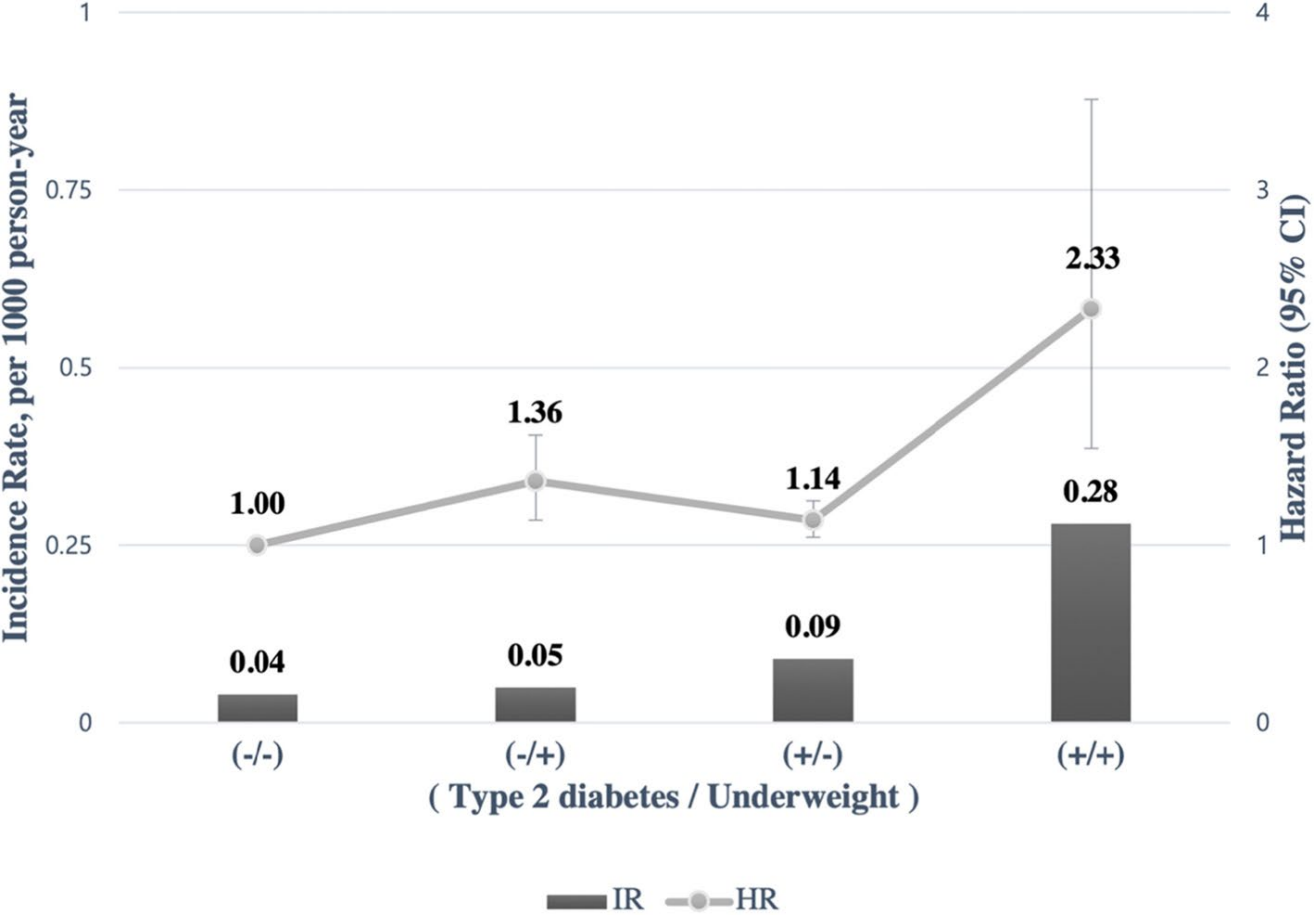
Association between underweight, type 2 diabetes and the development of laryngeal cancer. The combination of underweight and type 2 diabetes showed a higher risk of laryngeal cancer. The analysis was adjusted for age, sex, smoking, alcohol consumption, regular physical activity, income, type 2 diabetes, hypertension, dyslipidemia, chronic kidney disease, and anemia

The interaction between underweight and type 2 diabetes in the association with the risk of laryngeal cancer was shown in Fig. 2. Underweight and type 2 diabetes were synergistically associated with increased risk of laryngeal cancer. Individual with both underweight and type 2 diabetes showed the highest risk of laryngeal cancer (HR: 2.33, 95% CI: 1.54–3.51). The association between BMI and the risk of laryngeal cancer according to type 2 diabetes status was shown in Table 3. Compared to those who had a normal weight (BMI 18.5–22.9 kg/m^2^) but had no type 2 diabetes, those with a BMI < 18.5 kg/m^2^ and type 2 diabetes were at the highest risk for developing laryngeal cancer (HR: 2.02, 95% CI: 1.34–3.06).

**Table 3:** Hazard ratios for developing laryngeal cancer, according to the status of underweight and type 2 diabetes

	IR (per 1000 person-years)	HR (95% CI)		
		Model 1	Model 2	Model 3
BMI, kg/m^2^				
**Type 2 diabetes (−)**				
< 18.5	0.046	**1.31 (1.09–1.57)**	1.19 (0.99–1.42)	1.20 (1.00–1.44)
18.5–22.9	0.040	1 (Reference)	1 (Reference)	1 (Reference)
23–24.9	0.039	0.80 (0.73–0.88)	0.86 (0.78–0.94)	0.85 (0.77–0.93)
25–29.9	0.035	0.72 (0.65–0.79)	0.79 (0.72–0.86)	0.76 (0.69–0.84)
≥ 30	0.019	0.66 (0.49–0.88)	0.72 (0.53–0.96)	0.67 (0.50–0.90)
BMI, kg/m^2^				
**Type 2 diabetes (+)**				
< 18.5	0.275	**2.37 (1.57–3.58)**	**2.09 (1.38–3.15)**	**2.02 (1.34–3.06)**
18.5–22.9	0.135	**1.29 (1.11–1.48)**	**1.26 (1.09–1.45)**	**1.20 (1.04–1.39)**
23–24.9	0.104	1.02 (0.87–1.19)	1.07 (0.91–1.25)	1.00 (0.85–1.17)
25–29.9	0.071	0.81 (0.70–0.94)	0.87 (0.75–1.01)	0.80 (0.69–0.94)
≥ 30	0.058	1.16 (0.81–1.68)	1.25 (0.87–1.81)	1.13 (0.78–1.64)

Model 1 was adjusted for age, sex

Model 2 was adjusted for age, sex, income status, smoking, alcohol consumption, and physical activity

Model 3 was adjusted for age, sex, income status, smoking, alcohol consumption, physical activity, type 2 diabetes, hypertension, dyslipidemia, and anemia

*Abbreviations BMI* Body mass index, *CI* Confidence interval, *HR* Hazard ratio, *IR* Incidence rate (1000 person-year)

**Fig. 3:**
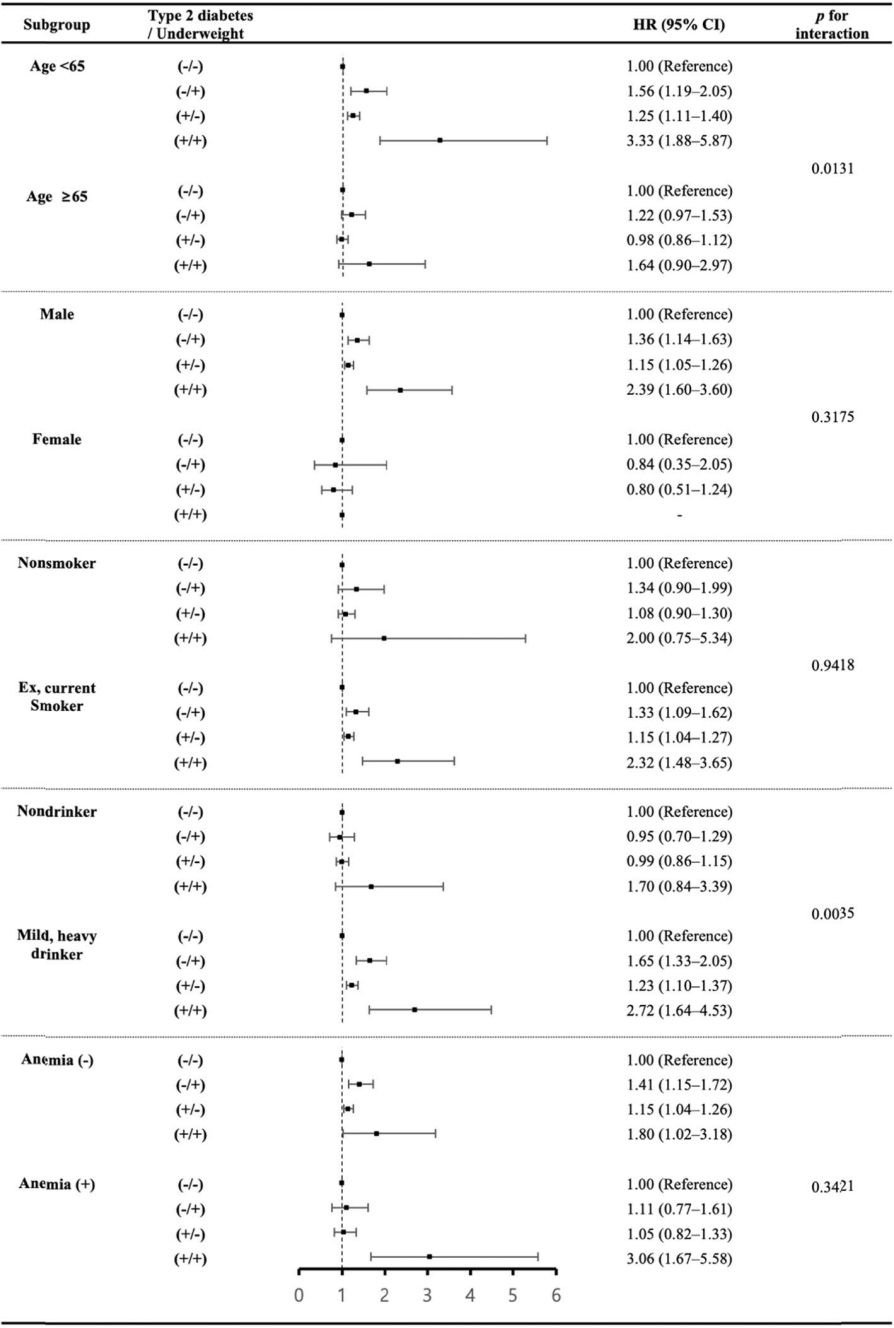
Forest plots including subgroup analyses for the association between participant characteristics and laryngeal cancer. Subgroup analyses were performed to investigate the associations between age, sex, smoking, alcohol consumption, and anemia and the development of laryngeal cancer according to the status of underweight and type 2 diabetes. The analysis was adjusted for age, sex, income, smoking, alcohol consumption, regular physical activity, hypertension, dyslipidemia, and anemia

Subgroup analyses were performed to determine whether the association between laryngeal cancer and underweight with type 2 diabetes varies according to participant characteristics (Fig. 3). The associations differed by age group (*p* for interaction, 0.01) and alcohol consumption (*p* for interaction, 0.004). Although the association between underweight, type 2 diabetes and laryngeal cancer remained significant in most subgroups, this association was more pronounced in subjects aged < 65 years (HR: 3.33, 95% CI: 1.88–5.87) and in those who were alcohol consumers (HR: 2.72, 95% CI: 1.64–4.53). Regarding the status of both underweight and type 2 diabetes, the HR (3.06, 95% CI; 1.67–5.58) of laryngeal cancer was increased in subjects with anemia, although the with and without anemia groups might not be significantly different (*p* for interaction, 0.34).

## Discussion

To the best of our knowledge, this is the first nationwide population-based study on the relationship between underweight, type 2 diabetes and laryngeal cancer. Our results showed that underweight was associated with increased risk of laryngeal cancer, and this risk was stronger for those with type 2 diabetes. Thus, the combination of underweight and type 2 diabetes might result in a synergistic effect on the development of laryngeal cancer.

Traditionally, smoking and some environmental factors have been suggested as risk factors for HNC. The well-known risk factors for laryngeal cancer include cigarette smoking and alcohol consumption, exposure to asbestos, polycyclic aromatic hydrocarbons (PAHs), and textile dust and dietary factors [26, 27]. Laryngopharyngeal reflux and human papilloma virus infection have also been suggested as possible risk factors [26–29]. Smoking has been shown to have a linear association with the development of laryngeal cancer, with a risk for smokers that is 10 to 15 times higher than the risk for nonsmokers [26]. It has been reported that the levels of DNA adduct formation by tobacco-specific nitrosamines (TSNA) and PAHs in smokers who have developed HNC are significantly higher [30, 31]. Since these aromatic DNA adducts are lipophilic, they can be distributed to adipose tissue [12]. Hence, low serum carcinogen levels can be found in patients with relatively high BMI [32]. This might explain why the risk of the development of laryngeal cancer is higher in underweight individuals.

In addition, there are several other hypotheses as to why the risk of HNC is relatively high in the group with low BMI. First, painful premalignant lesions could result in dysphagia, odynophagia, and taste and appetite changes, and thus, a decrease in caloric intake may cause weight loss [12, 13]. Another possibility is that underweight patients may have relatively low vitamin and micronutrient levels, which may increase the risk of HNC [13, 31]. In patients showing chronic undernutrition and micronutrient deficiency, cytokine reactions and the subsequent activation of the immune system are compromised, which may affect tumor–immune system interactions [33]. Third, low socioeconomic status, especially low educational attainment, low income and undernutrition, has been reported as a potential risk for HNC [13, 34]. In the present study, undernutrition and socioeconomic status were adjusted by controlling for the presence of anemia and income status, respectively. Although the status of vitamins and micronutrients was not clinically measured, our results might confirm that underweight was associated with increased risk of laryngeal cancer after adjusting for potential confounders.

Diabetes mellitus (DM) is one of the most common diseases worldwide. There are approximately 5.34 million diabetic patients among the 50 million people in Korea [35]. 90 ~ 95% of DM patients have type 2 disease that is characterized by years of insulin resistance and hyperinsulinemia preceding the development of hyperglycemia [36]. In particular, type 2 diabetes is a risk factor for cancer, particularly stomach, hepatocellular, hepatobiliary, pancreas, breast, ovarian, endometrial, kidney and colorectal cancers [7, 37, 38]. The possible connection between type 2 diabetes and cancer comprises hyperinsulinemia, hyperglycemia and fat-induced chronic inflammation [39]. High insulin levels inversely correlate with insulin-like growth factor binding protein (IGFBP)-1 and − 2 levels, which potentially leads to more free IGF-1 at the tissue and cellular levels. Binding of insulin to the insulin receptor primarily activates the phosphoinositide 3-kinase (PI3K)–tyrosine kinase B (Akt)–mammalian target of rapamycin (mTOR) signaling pathway [36]. Binding of IGF-1 and IGF-2 to IGF-1R stimulates the PI3K-Akt-mTOR and Ras-Raf-MAPK pathways [36, 40]. These pathways may lead to mitogenic signaling and increased tumor growth or progression. One study reported that overexpression of IGF1R-alpha protein could act as an independent predictor of relapse and survival in operable laryngeal cancer patients [41]. A recent meta-analysis indicated that type 2 diabetes is associated with an increased risk of HNC in East Asia and that the risk of oral cancer was most significantly increased among HNC patients [42]. However, conflicting results have been reported on whether type 2 diabetes is associated with increased risk of laryngeal cancer [28, 42, 43]. On the other hand, BMI has a strong relationship with type 2 diabetes. Lean patients with type 2 diabetes may have a tendency toward certain pathophysiological characteristics, notably less insulin resistance and poorer insulin secretory capacity. They would have β-cell dysfunction, which might be more marked than that seen in overweight patients [44]. Since some lean patients with type 2 diabetes may be more insulin sensitive, PI3K-Akt-mTOR pathway could be further stimulated by insulin and IGF-1, compared to in obese patients. Accordingly, underweight and type 2 diabetes were shown to be synergistically associated with increased risk of newly developed laryngeal cancer after adjusting for confounders including smoking, in this study.

We found that alcohol consumers who were underweight and had type 2 diabetes were more likely to develop laryngeal cancer than those who did not consume alcohol. Generally, alcohol consumption has been known to be associated with an approximately 2-fold increase in risk of laryngeal cancer [45]. The direct association between alcohol consumption and HNC risk has been extensively described and potential mechanisms have been proposed [46]. Ethanol is oxidized to alcohol acetaldehyde, which is a recognized carcinogen. Furthermore, nutritional deficiencies may occur in individuals with alcoholism. Alcohol exposure may also have a synergistic effect with other carcinogens. Alcohol consumption may be responsible for strengthening the relationship between underweight in the presence of type 2 diabetes and laryngeal cancer. Further studies are needed to determine whether serum levels of carcinogens are actually high in underweight alcoholic patients.

It is unclear why the group under 65 years showed a stronger relationship between underweight, type 2 diabetes and laryngeal cancer. Long-term exposure to smoking and alcohol consumption is well known as a major risk factor for aerodigestive malignancies, including laryngeal cancer. It may be a lower attributable fraction for smoking and drinking in the group under 65 years compared to the group above 65 years. It is likely explained by some molecular- or genetic-level abnormalities. Dysregulation of the Akt pathway occurred in nonsmoker HNC patients, but did not occur in all of the smokers studied [47]. As mentioned above, the PI3K-Akt-mTOR pathway is very important for carcinogenesis associated with IGF-1. Further studies are needed to confirm whether this same signaling pathway supports the results of subgroup analysis of younger patients. In addition, based on the results of this study, more observation and education on laryngeal cancer prevention programs are needed in adults under the age of 65 years who are underweight and have type 2 diabetes. They also might need to undergo appropriate laryngeal cancer screenings to promote primary prevention and early detection.

There are some limitations in this study. First, we used an observational design; therefore, inferences of causality should not be made regarding the association of underweight and type 2 diabetes with the development of laryngeal cancer. Second, we had limited information to define a pathologic classification of tumor and tumor stage. Third, our cohort comprised primarily Korean participants, and our results need to be validated in a more diverse population of patients. Fourth, the value of BMI was collected one time at health check-ups. Since BMI is a changeable variable, it may be different when laryngeal cancer is diagnosed. Finally, we could not directly assess the severity of type 2 diabetes or glycemic control status and did not have reliable information on the diverse combinations of medications used.

Despite these limitations, this is a nationally representative, population-based long-term cohort study evaluating the association of underweight and type 2 diabetes as risk factors for laryngeal cancer. A future study could help elucidate these two risk factors for laryngeal cancer outcomes.

## Conclusions

We found that underweight was an independent risk factor for developing laryngeal cancer in a nationwide Korean general population-based cohort study. This study showed that underweight and type 2 diabetes were synergistically associated with a higher risk of laryngeal cancer. The risk of laryngeal cancer was stronger for those younger than 65 years old and alcohol consumers. Based on the results of this study, it might be necessary to more actively screen for laryngeal cancer in adults with underweight and type 2 diabetes, and clinical attention should be given to smoking cessation and to the elimination of environmental risk factors in this high-risk group.

## Data Availability

The data that support the findings of this study are available on request from the corresponding author. The data are not publicly available due to privacy or ethical restrictions.

## Abbreviations

Akt: Tyrosine kinase B
BMI: Body mass index
CI: Confidence interval
CKD: Chronic kidney disease
DBP: Diastolic blood pressure
DM: Diabetes mellitus
DNA: Deoxyribonucleic acid
eGFR: Estimated glomerular filtration rate
HDL-C: High-density lipoprotein cholesterol
HNC: Head and neck cancer
HR: Hazard ratio
ICD-10: International Classification of Diseases, 10th version
IGF-1: Insulin-like growth factor 1
IGF1R: Insulin like growth factor 1 receptor
IGFBP: Insulin like growth factor binding protein
IR: Incidental rate
IRB: Institutional Review Board
KHIDI: Korea Health Industry Development Institute
MAPK: Mitogen activated protein kinase
MDRD: Modification of diet in renal disease
mTOR: Mammalian target of rapamycin
NHIS: National Health Insurance Service
PAH: Polycyclic aromatic hydrocarbons
PI3K: Phosphoinositide 3-kinase
SBP: Systolic blood pressure
TSNA: Tobacco-specific nitrosamines
WC: Waist circumference
WHO: World Health Organization
